# Lobular carcinoma in situ of the breast is not caused by constitutional mutations in the E-cadherin gene

**DOI:** 10.1054/bjoc.1999.0965

**Published:** 2000-01-18

**Authors:** N Rahman, J G Stone, G Coleman, B Gusterson, S Seal, A Marossy, S R Lakhani, A Ward, A Nash, A McKinna, R A'Hern, M R Stratton, R S Houlston

**Affiliations:** Section of Cancer Genetics, Section of Cell Biology and Experimental Pathology, Institute of Cancer Research, Sutton, SM2 5NG, UK; Breast Unit, Department of Computing, Royal Mardsen Hospital, Sutton, SM2 5NG, UK

**Keywords:** LCIS, germline, E-cadherin mutations

## Abstract

Lobular carcinoma in situ (LCIS) is an unusual histological pattern of non-invasive neoplastic disease of the breast occurring predominantly in women aged between 40 and 50 years. LCIS is frequently multicentric and bilateral, and there is evidence that it is associated with an elevated familial risk of breast cancer. Although women with LCIS suffer an increased risk of invasive breast disease, this risk is moderate suggesting that LCIS may result from mutation of a gene or genes conferring a high risk of LCIS, but a lower risk of invasive breast cancer. The high frequency of somatic mutations in E-cadherin in LCIS, coupled with recent reports that germline mutations in this gene can predispose to diffuse gastric cancer, raised the possibility that constitutional E-cadherin mutations may confer susceptibility to LCIS. In order to explore this possibility we have examined a series of 65 LCIS patients for germline E-cadherin mutations. Four polymorphisms were detected but no pathogenic mutations were identified. The results indicate that E-cadherin is unlikely to act as a susceptibility gene for LCIS. © 2000 Cancer Research Campaign


British Journal of Cancer (2000) **82**(3), 568–570
© 2000 Cancer Research Campaign
DOI: 10.1054/ bjoc.1999.0965, available online at http://www.idealibrary.com on IDEAL®

### Lobular carcinoma in situ of the breast is not caused by constitutional mutations in the E-cadherin gene

**N Rahman[1,*], JG Stone[1,*], G Coleman[1], B Gusterson[2], S Seal[1], A Marossy[1], SR Lakhani[1†], A Ward[2], A Nash[3], A McKinna[3], R A'Hern[4], MR Stratton[1] and RS Houlston[1]**

[1]Section of Cancer Genetics and [2]Section of Cell Biology and Experimental Pathology, Institute of Cancer Research, Sutton, SM2 5NG, UK; [3]Breast Unit, and [4]Department of Computing, Royal Mardsen Hospital, Sutton, SM2 5NG, UK

**Summary** Lobular carcinoma in situ (LCIS) is an unusual histological pattern of non-invasive neoplastic disease of the breast occurring predominantly in women aged between 40 and 50 years. LCIS is frequently multicentric and bilateral, and there is evidence that it is associated with an elevated familial risk of breast cancer. Although women with LCIS suffer an increased risk of invasive breast disease, this risk is moderate suggesting that LCIS may result from mutation of a gene or genes conferring a high risk of LCIS, but a lower risk of invasive breast cancer. The high frequency of somatic mutations in E-cadherin in LCIS, coupled with recent reports that germline mutations in this gene can predispose to diffuse gastric cancer, raised the possibility that constitutional E-cadherin mutations may confer susceptibility to LCIS. In order to explore this possibility we have examined a series of 65 LCIS patients for germline E-cadherin mutations. Four polymorphisms were detected but no pathogenic mutations were identified. The results indicate that E-cadherin is unlikely to act as a susceptibility gene for LCIS. © 2000 Cancer Research Campaign

**Keywords:** LCIS; germline; E-cadherin mutations

Lobular carcinoma in situ of the breast (LCIS) is a relatively rare disease (incidence rate: in Europe 9/100 000, USA between 15 and 17/100 000) (Levi et al, 1997) with a distinctive histological appearance characterized by masses of loosely arranged cells with round, monotonous hyperchromatic nuclei that distend acini of the lobular unit. Mitoses, necrosis and cellular anaplasia are usually absent (Foote et al, 1941; Frykberg et al, 1987; Beute et al, 1991). In contrast to ductal carcinoma in situ (DCIS), the disease is often multicentric within one breast, and in half or more cases is bilateral (Ottesen et al, 1993; Millikan et al, 1995). Over 80% of patients with LCIS are diagnosed between 40 and 50 years of age, usually as an incidental finding in a biopsy taken for other palpable or mammography-detected benign or malignant lesions (Bartow et al, 1987; Frykberg et al, 1987; Beute et al, 1991).

LCIS confers an elevated risk of invasive cancer. Over the 25 years following diagnosis, approximately one-fifth of LCIS cases will develop invasive cancer. Many of these occur in young women, and the risk of breast cancer in LCIS is increased tenfold (Page et al, 1991; Ottesen et al, 1993; Milikan et al, 1995). Invasive cancers are equally likely to occur in the contralateral breast as in the breast known to carry LCIS (Millikan et al, 1995). This is in contrast to partially resected DCIS in which the invasive cancer usually develops in the same quadrant of the same breast. Approximately half of invasive cancers developing upon a background of LCIS are lobular in histological type, the remainder being a mixture of ductal, tubular and others (Page et al, 1991; Ottesen et al, 1993).

*Received 3 December 1998*
*Revised 26 May 1999*
*Accepted 22 July 1999*

*Correspondence to:* RS Houlston

The biological nature of LCIS and its relationship to invasive cancers is controversial. The multicentricity of the disease has led some authors to propose that it is a hyperplastic rather than a neoplastic process. Some authorities regard LCIS as a risk indicator for invasive cancer or a morphological marker of a carcinogenic stimulus, and do not believe that the cancer itself arises from the abnormal LCIS cells. An alternate view, which is generally accepted for DCIS, is that LCIS cells are intermediates in the progression to invasive cancer.

The pattern of early age of onset and multicentricity of neoplasms is reminiscent of heritable cancer predisposition syndromes, and suggests that LCIS may result from an inherited susceptibility. This hypothesis is supported by data showing that foci of LCIS are likely to be clonal (Lakhani et al, 1995). Furthermore, there is evidence from systematic studies that both LCIS and invasive lobular carcinoma are associated with higher familial risks of breast cancer than other histological types (Claus et al, 1993; Cannon-Albright et al, 1994). LCIS is not a manifestation of *BRCA1* or *BRCA2* mutations (BCLC, 1997) and therefore may be an indicator of a previously unrecognized cancer predisposition syndrome, in which the penetrance for invasive cancer is relatively low.

There are no known genes that confer susceptibility to LCIS. However, there is a-priori evidence suggesting that E-cadherin is a strong candidate for an LCIS predisposition gene. E-cadherin is a transmembrane adhesion protein with a central role in the maintenance of the normal architecture and function of epithelial cells (Takeichi, 1995). Over 400 tumours from ten different tissue types have been screened for E-cadherin mutations (Berx et al, 1998).

*Contributed equally. †Present address: Department of Histopathology, University College, London.

Somatic mutations occur frequently in two histological subtypes: diffuse gastric carcinomas and lobular breast cancers. In lobular breast carcinomas, the E-cadherin mutations generally result in premature truncation of translation and are usually accompanied by loss of the wild-type allele (Berx et al, 1995, 1996). This suggests that E-cadherin acts as a tumour suppressor gene. In LCIS, E-cadherin expression is almost always absent (Moll et al, 1993), and somatic E-cadherin mutations together with loss of heterozygosity (LOH) of the wild-type allele have been identified (Vos et al, 1997). In two breast cancers, the same mutation was identified in the LCIS and invasive components, supporting the theory that LCIS is an invasive precursor (Vos et al, 1997). In contrast, somatic E-cadherin mutations have not been reported in either DCIS or invasive ductal breast carcinomas and E-cadherin expression is not absent in these neoplasms (Vos et al, 1997; Berx et al, 1998). Loss of E-cadherin has been demonstrated in LCIS adjacent to E-cadherin-positive invasive lobular cancers (de Leeuw et al, 1997) indicating that loss of E-cadherin is an important early step in the formation of LCIS. To our knowledge, the presence of constitutional E-cadherin mutations in individuals with LCIS has not been investigated. However, constitutional E-cadherin mutations that predispose to familial diffuse gastric cancer have been identified (Gayther et al, 1998; Guilford et al, 1998). In order to examine whether constitutional alterations in E-cadherin predispose to LCIS we have analysed blood samples from 65 patients with LCIS for germline mutations in the gene.

### PATIENTS AND METHODS

### Patients

All individuals with a histologically proven diagnosis of LCIS that attended the Royal Marsden Hospital between 1971 and 1996 were invited to participate. Samples were obtained with informed consent and local ethical review board approval. EDTA-venous blood samples were obtained from 65 patients. DNA was extracted using a standard sucrose lysis protocol.

### Methods

The full coding sequence and splice junctions of E-cadherin were screened for mutations using conformational specific gel electrophoresis (CSGE) (Ganguly et al, 1993). Published oligonucleotide sequences were used to amplify each exon of the E-cadherin gene (including splice sites) by polymerase chain reaction (PCR) (Berx et al, 1995). All samples with bandshifts detected by CSGE were sequenced in duplicate and in forward and reverse orientations after re-amplification of the appropriate exon from genomic DNA in the PCR. Purified PCR products were sequenced using ABI Ready Reaction Dye Terminator Cycle Sequencing Kit and the ABI 377 Prism sequencer.

### RESULTS

DNA from 65 patients with a histologically proven diagnosis of LCIS was obtained. None of the patients had invasive cancer at the time of diagnosis of LCIS. The clinical details of the patients are shown in Table 1. Seventeen of the patients had bilateral disease and 21 also had a diagnosis of DCIS. Twenty of the patients had a first-degree relative affected with invasive breast cancer, but only one had a family history highly suggestive of the inheritance of a dominantly acting breast cancer susceptibility gene.

**Table 1** Ages and clinical characteristics of the 65 patients with LCIS

| | |
|---|---|
| Age (years) | |
| Mean (± s.d.) | 48 (7.7) |
| Range | 26–71 |
| Breast disease | |
| Bilateral LCIS | 17/65 (26%) |
| Co-existing DCIS | 21/65 (32%) |
| Family history of breast cancer | |
| Affected first-degree relative | 20/65 (31%) |
| Dominant pedigree | 1/65 (2%) |

**Table 2** Summary of E-cadherin gene variations detected

| No. of patients | Codon no. | Amplicon | Polymorphism |
|---|---|---|---|
| 1 | 115 | Exon 3 | ACG (Thr) to ACA (Thr) |
| 4 | 632 | Exon 12 | CAC (His) to CAT (His) |
| 28 | 692 | Exon 13 | GCC (Ala) to GCT (Ala) |
| 2 | 751 | Exon 14 | AAC (Asn) to AAT (Asn) |

The full coding sequence and splice junctions of E-cadherin were screened for mutations in all samples. No pathogenic mutations were identified in any of the patients screened. Four polymorphic variants were detected in 29 of the patients (Table 2). All were synonymous substitutions and have been previously reported (Berx et al, 1998).

### DISCUSSION

We have obtained DNA from 65 individuals with LCIS. Thirty-two per cent of the patients studied had a family history of invasive breast cancer suggesting that LCIS confers a fourfold increase in breast cancer risk in first-degree relatives. Twenty-six per cent of patients had bilateral disease. These data are concordant with the hypothesis that a proportion of LCIS results from inherited predisposition and suggests that a LCIS susceptibility gene may also confer an elevated risk of invasive breast cancer.

E-cadherin is mutated somatically at high frequency in LCIS, invasive lobular breast cancer and diffuse gastric cancer (Berx et al, 1998). Constitutional predisposing E-cadherin mutations have recently been detected in familial gastric cancer pedigrees (Gayther et al, 1998; Guildford et al, 1998). We have examined lymphocyte DNA from 65 individuals with LCIS, for germline alterations in E-cadherin. No disease-causing alterations were identified. This suggests that constitutional mutations in E-cadherin do not confer susceptibility to LCIS.

We cannot exclude the possibility that a minority of mutations have been missed, or cannot be detected by a PCR-based approach. However, under test conditions we have found this technique can detect all small insertions and deletions and 90% of single-base substitutions. Confirmation of the efficiency of this technique is that we were able to demonstrate a number of single-base substitution polymorphisms within the gene. Therefore it is unlikely that we have failed to detect any coding mutations.

It is theoretically possible that constitutive mutations in E-cadherin are responsible for a few LCIS cases. However, based on the number of patients we have examined we can conclude with 95% probability that germline variation in E-cadherin does not account for more than 4% of cases of LCIS.

The high frequency of somatic mutations in the E-cadherin gene in LCIS coupled with the recent finding that germline mutations in the gene can predispose to diffuse gastric cancer suggested that constitutional E-cadherin mutations might confer susceptibility to LCIS. The results presented indicate that this is very unlikely and that the majority of LCIS cases do not result from germline mutations in E-cadherin. However, the elevated incidence of bilateral LCIS and of invasive breast cancer in relatives, supports the hypothesis that a proportion of LCIS results from genetic susceptibility. The identity of this susceptibility gene is unknown, but may also be a low penetrance invasive breast cancer susceptibility gene.

### Note added in proof

A frameshift mutation in exon 3 of E-cadherin has recently been reported in a patient with LCIS who had a strong family history of gastric cancer (Keller et al, 1999).

### ACKNOWLEDGEMENTS

We thank the Cancer Research Campaign for support and the patients for their participation in this study. NR is a MRC Clinical Training Fellow. Sequencing was conducted in the Jean Rook Sequencing Laboratory within the Institute of Cancer Research, which is supported by BREAKTHROUGH Breast Cancer, charity 328323.

### REFERENCES

Bartow SA, Pathak DR, Black WC, Key CR and Teaf SR (1987) Prevalence of benign, atypical, and malignant breast lesions in populations at different risks for breast cancer. A forensic autopsy study. *Cancer* **60**: 2751–2760

Beute BJ, Kalisher L and Hutter RVP (1991) Lobular carcinoma in situ of the breast: clinical, pathological and mammographic features. *Am J Radiol* **157**: 257–265

Berx G, Cleton-Jansen AM, Nollet F, de Leeuw WJ, van de Vijver M, Cornelisse C and van Roy F (1995) E-cadherin is a tumour/invasion suppressor gene mutated in human lobular breast cancer. *EMBO J* **14**: 6107–6115

Berx G, Cleton-Jansen AM, Strumane K, de Leeuw WJ, Nollet F, van Roy F and Cornelisse C (1996) E-cadherin is inactivated in a majority of invasive human lobular breast cancer by truncation mutations throughout its extracellular domain. *Oncogene* **13**: 1919–1925

Berx G, Becker KF, Hofler H and van Roy F (1998) Mutations of the human E-cadherin (CDH1) gene. *Hum Mutat* **12**: 226–237

Breast Cancer Linkage Consortium (1997) Pathology of familial breast cancer: differences between breast cancers in carriers of BRCA1 or BRCA2 mutations and sporadic cases. *Lancet* **249**: 1505–1510

Cannon-Albright L, Thomas A, Goldgar DE, et al (1994) Familiality of cancer in Utah. *Cancer Res* **54**: 2378–2385

Claus EB, Risch N and Thompson WD (1993) Relationship between breast histopathology and family history of breast cancer. *Cancer* **71**: 147–153

De Leeuw WJ, Berx G, Vos CB, Peterse JL, Van de Vijver MJ, Litvinov S, Van Roy F, Cornelisse CJ and Cleton-Jansen AM (1997) Simultaneous loss of E-cadherin and catenins in invasive lobular breast cancer and lobular carcinoma in situ. *J Pathol* **183**: 404–411

Foote FW and Stewart FW (1941) Lobular carcinoma in situ: a rare form of mammary cancer. *Am J Pathol* **17**: 491–495

Frykberg ER, Santiago F, Betsill WL and O'Brien PH (1987) Lobular carcinoma in situ of the breast. *Surg Gynecol Obstet* **164**: 285–301

Ganguly A, Rock MJ and Prockop DJ (1993) Conformation-sensitive gel electrophoresis for rapid detection of single-base differences in double-stranded PCR products and DNA fragments: evidence for solvent-induced bends in DNA heteroduplexes. *Proc Natl Acad Sci USA* **90**: 10325–10329

Gayther SA, Gorringe KL, Ramus SJ, Huntsman D, Roviello F, Grehan N, Machado JC, Pinto E, Seruca R, Halling K, MacLeod P, Powell SM, Jackson CE, Ponder BA and Caldas C (1998) Identification of germ-line E-cadherin mutations in gastric cancer families of European origin. *Cancer Res* **58**: 4086–4089

Guilford P, Hopkins J, Harraway J, McLeod, M, McLeod N, Harawira P, Taite H, Scoular R, Miller A and Reeve AE (1998) E-cadherin germline mutations in familial gastric cancer. *Nature* **392**: 402–405

Keller G, Vogelsong H, Becker I, Hutter J, Ott K, Candidus S, Grundei T, Becker KF, Mueller J, Siewert JR and Hofler H (1999) Diffuse type gastric and lobular breast carcinoma in a familial gastric cancer patient with an E-cadherin germline mutation. *Am J Pathol* **155**: 337–342

Lakhani SR, Collins N, Sloane JP and Stratton MR (1995) Loss of heterozygosity in lobular carinoma in situ of the breast. *J Clin Pathol* **48**: M74–M78

Levi F, Te VC, Randimbison L and La Vecchia C (1997) Trends of in situ carcinoma of the breast in Vaud, Switzerland. *Eur J Cancer* **33**: 903–906

Millikan R, Dressler L, Geradts J and Graham M (1995) The need for epidemiologic studies of in-situ carcinoma of the breast. *Breast Cancer Res Treat* **35**: 65–77

Moll R, Mitze M, Frixen UH and Birchmeier W (1993) Differential loss of E-cadherin expression in infiltrating ductal and lobular carcinomas. *Am J Pathol* **143**: 1731–1742

Otteson GL, Graverson HP, Blichert-Toft M, Zedeler K and Andersen JA (1993) Lobular carcinoma in situ of the female breast. *Am J Surg Pathol* **17**: 14–21

Page DL, Kidd TE, Dupont WC, Simpson JF and Rogers LL (1991) Lobular neoplasia of the breast: higher risk for subsequent invasive cancer predicted by more extensive disease. *Hum Pathol* **22**: 1232–1239

Takeichi M (1995) Morphogenetic roles of classical cadherins. *Curr Opin Cell Biol* **7**: 619–627

Vos CB, Cleton-Jansen AM, Berx G, de Leeuw WJ, ter Haar NT, van Roy F, Cornelisse CJ, Peterse JL and van de Vijver MJ (1997) E-cadherin inactivation in lobular carcinoma in situ of the breast: an early event in tumorigenesis. *Br J Cancer* **76**: 1131–1133

*© 2000 Cancer Research Campaign*
